# Is percutaneous dilational tracheostomy genuinely performed without the need for routine bronchoscope guidance?

**DOI:** 10.1186/s13054-026-05997-9

**Published:** 2026-04-07

**Authors:** Hao Qin, Wei Zhang

**Affiliations:** 1 Department of Respiratory and Critical Care Medicine, the First affiliated hospital of Second Military Medical University, Shanghai, 200433 P. R. China; 2 168 Changhai Road, Yangpu, Shanghai, 200433 China

## To the Editor

Nachshon et al. conducted a study [1] that confirmed bronchoscopy during percutaneous dilational tracheostomy (PDT) does not reduce major complications or mortality, but it does increase procedure duration, complexity, and costs. For experienced practitioners, performing PDT without bronchoscopy is safe and potentially more efficient. However, some questions remain to be clarified.

Firstly, the authors noted that bronchoscopy can affect gas exchange, potentially leading to hypercapnia, hypoxemia, and increased intracranial pressure. Yet, the study found no significant difference in desaturation rates between the groups (8% vs. 7%, *p* = 0.84), and blood gas results weren’t compared. Could it be that airway secretions were completely removed through bronchoscopy, but not without it, leading to similar desaturation levels in both groups? Additionally, in the study design, PDT was performed directly with a laryngoscope, not a bronchoscope. How was this procedure carried out? Was there a specific process involved? And did using laryngoscopy add to the costs? Patients with uncontrolled coagulopathy were excluded; what about the coagulation status of these patients? Since critically ill ICU patients often have coagulation issues, should such patients be excluded? The abbreviation for percutaneous dilatational tracheostomy is PDT, is PCT wrong?

Secondly, three patients (1%) died within 24 h, including one death directly linked to the procedure. Could the absence of bronchoscopy have played a role? The data does not specify patients’ BMI values. Was there a difference in BMI between the groups? Such a difference might influence the outcomes. Regarding the operational time issue, the two studies cited in the discussion yield different results. Does this suggest that operation time depends on many factors? For instance, Gobatto ALN et al. [2] reported an operation time of 13 min in the bronchoscope-guidance group.

Finally, in Table 3, how can we explain that experienced doctors in the bronchoscopy-assisted group took longer from sedation to skin incision? In Supplementary Table 4, without bronchoscopy, experienced physicians showed a higher rate of major complications (1.1% vs. 11.1%, *p* = 0.01). Why might that be? Although the author explains it, it still doesn’t seem to align with common sense.

The bronchoscope is a valuable visualization tool during tracheostomy in critically ill patients. It generally guides the withdrawal of tracheal intubation above the puncture site to ensure proper placement, avoiding damage to the cricoid cartilage if positioned too high or erosion to the innominate artery if too low. It also confirms whether the puncture point is at or close to the 12 o’clock position along the anterior midline. Regarding puncture direction, deviations from the midline can cause the cannula tip to adhere to the wall. Once the tracheotomy tube is inserted, the bronchoscope confirms the cannula’s placement and aids in aspirating secretions. Passing through the nose enables the assessment of abnormalities in the proximal trachea, subglottic space, and vocal cords. Special attention should be paid to the posterior commissure and supraglottic structures, which may lead to late complications [3, 4]. Long-term intubation increases these risks [5]. Compared to these potential issues, extending the PDT by a minute might be a minor concern.

In conclusion, PDT time may depend on the operator’s experience, daily habits, and teamwork skills. Excellent teamwork can ensure that bronchoscopic PDT does not necessarily extend the procedure time and may decrease early and late airway-related complications. As a single-center study, the evidence is insufficient to draw definitive conclusions.

## Data Availability

No datasets were generated or analysed during the current study.

## Abbreviations

PDT: Percutaneous dilational tracheostomy
BMI: Body Mass Index
